# Randomized controlled trial of S-1 maintenance therapy in metastatic esophagogastric cancer – the multinational MATEO study

**DOI:** 10.1186/s12885-017-3497-9

**Published:** 2017-07-31

**Authors:** Georg-Martin Haag, Gertraud Stocker, Julia Quidde, Dirk Jaeger, Florian Lordick

**Affiliations:** 1 Department of Medical Oncology, National Center for Tumor Diseases, University Hospital Heidelberg, Im Neuenheimer Feld 460, D-69120 Heidelberg, Germany; 2 University Cancer Center Leipzig (UCCL), University Hospital Leipzig, Liebigstrasse 20, D–04103 Leipzig, Germany; 3 II. Department of Internal Medicine (Oncology/Haematology), University Medical Center Hamburg-Eppendorf, Martinistraße 52, D-20246 Hamburg, Germany

**Keywords:** Gastric cancer, Esophagogastric cancer, Adenocarcinoma, S-1, Chemotherapy, Maintenance therapy

## Abstract

**Background:**

The optimal duration of firstline chemotherapy in metastatic esophagogastric cancer is unknown. In most clinical trials therapy was given until tumour progression or limiting toxicity. Maintenance concepts aiming to prolong the duration of response and maintain quality of life have been established in other tumour types but not in esophagogastric cancer. S-1 is an oral fluoropyrimidine with proven efficacy in metastatic esophagogastric cancer.

**Methods:**

The Maintenance Teysuno® (S-1) in esophagogastric cancer (MATEO) trial is a multinational, randomized phase II study that explores the role of S-1 maintenance therapy in Her-2 negative, advanced esophagogastric adenocarcinoma. After a 12-week firstline platinum-fluoropyrimidine-based chemotherapy patients without tumour progression are randomized in a 2:1 allocation to receive S-1 alone or continue with the same regimen as during the primary period. The primary endpoint is overall survival. Secondary endpoints include safety and toxicity, progression-free survival and quality of life.

Correlative biomarker analyses focus on the identification of a subgroup of patients with a prolonged benefit from S-1 based maintenance therapy.

**Discussion:**

MATEO will be the first trial to define the role of a S-1 based maintenance therapy in patients having received a platinum-based firstline chemotherapy.

**Trial registration:**

NCT02128243 (date of registration: 29–04-2014).

## Background

Gastric cancer is the third most common cause of cancer related death worldwide. Every year around 725,000 people die due to gastric cancer [1]. A large geographical variability of the prevalence in Asia, Europe and America but also within the continents is observed.

In the beginning of the twentieth century most gastric cancers were located in the distal parts of the stomach. While during the last decades the overall incidence of gastric cancer has declined, especially in Western countries, a strong increase in tumours located at the esophagogastric junction (adenocarcinoma of the esophagogastric junction, AEG) occurred [2, 3].

Due to a lack of early symptoms and no effective screening programs most gastric cancers and AEGs are diagnosed in irresectable advanced or metastatic stages in Western countries where even in localized stages long term survival is below 50% [4, 5]. Relapse rates after surgical treatment with or without perioperative treatment remain high and a majority of patients will eventually die due to recurrent or metastatic disease.

In patients with metastatic disease, palliative chemotherapy remains the mainstay of treatment. This therapy aims to prolong survival and to improve or maintain quality of life by ameliorating tumour-related symptoms.

Superiority in terms of overall survival compared with best supportive care was shown in a meta-analysis published by Wagner and colleagues [6]. Combination chemotherapy showed significant better outcomes in comparison with monotherapy.

Until now only Her-2 overexpressing tumours, a subgroup of ~16% of esophagogastric cancers, are eligible for biologically targeted therapy in the firstline treatment of advanced disease. The combination of trastuzumab with cisplatin and a fluoropyrimidine has shown superiority in the phase III *Trastuzumab for Gastric Cancer* (ToGa) trial [7]. Following progression to firstline, the anti-VEGFR2 (vascular endothelial growth factor receptor 2) monoclonal antibody ramucirumab has proven efficacy either as monotherapy or in combination with paclitaxel [8, 9].

For patients with Her-2 negative gastric cancers undergoing firstline treatment for metastatic disease no approved targeted agents are available, thus chemotherapy remains the treatment of choice.

Platinum compounds combined with fluoropyrimidines are the mainstay of polychemotherapy for Her-2 negative esophagogastric cancer having shown superiority over older regimens containing methotrexate and etoposide [10, 11].

For several years the ECF regimen, consisting of epirubicin, cisplatin and 5-FU (5-fluorouracil), has been considered to be a reference regimen in the metastatic setting. The REAL2 phase III trial has shown non-inferiority of oxaliplatin in comparison with cisplatin and capecitabine in comparison with 5-FU. In addition, overall survival was modestly longer in the EOX group (epirubicin, oxaliplatin, capecitabine) compared with the ECF group. Therefore, EOX is nowadays considered a standard regimen for metastatic esophagogastric cancer [12].

Adding a taxane to cisplatin and 5-FU (DCF regimen) significantly improved the response rate, time-to-tumour-progression (TTP) and overall survival (OS) in the TAX 325 trial [13]. Although the grade 3–4 toxicity in terms of diarrhoea, neutropenia and febrile neutropenia was significantly higher, assessment of quality of life has shown a prolonged time to definitive worsening of the performance status in the investigational arm [14]. Due to the increased toxicity associated with the DCF regimen, several study groups have performed phase II trials assessing modifications of dosing and scheduling. As a result, modified DCF [15], FLOT [16], the Gastro-Tax regimen [17] and others are now being used in daily practice, although they have not been validated in randomized phase III trials.

The optimal duration of firstline chemotherapy in advanced esophagogastric cancer is unknown. In most phase III trials chemotherapy was given until tumour progression or the occurrence of inacceptable toxicity [18].

As an exception, in the REAL2 trial, chemotherapy was given for a maximum of 8 cycles, each with duration of 21 days [12]. In the FLAGS trial (cisplatin combined with S-1 or 5-FU) combination chemotherapy was given for a maximum of 6 four-weekly cycles (24 weeks), followed by fluoropyrimidine monotherapy [19].

Dose reductions of chemotherapeutical components due to toxicity are not uncommon in patients receiving two- or three-drug combinations. For example, dose reductions were necessary in 35–42% of all patients in the REAL2 trial [12].

So far no randomized trials have been performed to determine the optimal duration of firstline chemotherapy in metastatic esophagogastric cancer. In clinical routine, firstline chemotherapy is mostly given until tumour progression or inacceptable toxicity occurs.

Prolonged periods of chemotherapy in responding patients often lead to increased cumulative toxicity. Therefore, concepts of therapy de-escalation and the idea of a maintenance therapy have been addressed in several tumour entities.

The goal of a maintenance therapy is to consolidate the tumour regression that has been achieved during the first months of combination chemotherapy (primary period) and at the same time to avoid excessive toxicity which often occurs with prolonged polychemotherapy. Maintenance concepts have been established in lung cancer and in colorectal cancer [20, 21].

In this trial S-1 was chosen for assessing the role of maintenance therapy in advanced esophagogastric cancer. S-1 is an oral fluoropyrimidine consisting of tegafur (a prodrug that is converted to fluorouracil, mainly in liver microsomes but also in tumour tissue), gimeracil (an inhibitor of dihydropyrimidine dehydrogenase, which degrades 5-FU), and oteracil (which inhibits the phosphorylation of 5-FU in the gastrointestinal tract, thereby reducing the toxic effects of 5-FU in the intestinum).

S-1 administered in combination with cisplatin has been recently approved in Europe for the treatment of advanced gastric cancer based on the results of the S1301/FLAGS study [19]. In this study, non-inferiority of cisplatin/S-1 in comparison with cisplatin/5-FU in terms of overall survival was shown. Cisplatin/S-1 displayed significantly fewer and less severe toxicity than cisplatin/5-FU.

### Objectives

The primary objective of the MATEO trial is to assess the relative efficacy of S-1 maintenance therapy vs. continuation of polychemotherapy after a primary phase in patients with metastatic esophagogastric cancer in terms of overall survival.

Secondary objectives include the comparison of S-1 maintenance therapy vs. continuation of polychemotherapy after primary therapy with respect to safety/toxicity, progression-free survival and quality of life. Translational analyses focus on identifying a molecularly defined subgroup of patients with a sustained benefit from S-1 based maintenance therapy.

## Methods/design

The MATEO trial is an open-label, multi-centre, controlled randomized, parallel-group phase II non-inferiority trial in patients with metastatic esophagogastric cancer. A randomized control group was incorporated into the design since reliance on historic data alone for assessing the relative efficacy of de-escalation with S-1 and continuation of polychemotherapy is not appropriate. 297 patients in six European countries are planned to be randomized.

### Trial population

Patients with metastatic, Her-2 negative esophagogastric adenocarcinoma will be registered before or after the initiation of a firstline chemotherapy regimen. Patients who have previously been treated with curative surgery with or without perioperative (neoadjuvant and/or adjuvant treatment) are eligible if primary treatment for metastatic disease starts at least 6 months after the end of perioperative treatment. Details of the in- and exclusion criteria can be found in Table 1.

**Table 1:** Inclusion and exclusion criteria

Inclusion criteria	Exclusion criteria
1. Signed written informed consent incl. participation in translational research 2. Male or female patient 18 years or older 3. Histologically confirmed metastatic or locally advanced unresectable gastric adenocarcinoma or adenocarcinoma of the esophagus or the esophagogastric junction (Her-2/neu negative or with unknown Her-2/neu status) 4. Adjuvant/neoadjuvant or perioperative chemotherapy or (chemo-)radiotherapy must have been finished at least 6 months before start of the induction therapy 5. For patients enrolled before induction therapy: No previous systemic treatment (i.e. chemotherapy) for metastatic disease 6. For patients enrolled after induction therapy: Having finished a three-months induction therapy (6 cycles of a bi-weekly regimen, 4 cycles of a three-weekly regimen or 3 cycles of a four-weekly regimen) without tumour progression or limiting toxicity 7. ECOG Performance Score 0–1 (Karnofsky Performance status > = 80%) 8. Ability for oral intake of the study drug, patients with tumour-related problems with oral intake might be registered if the symptom is expected to be improved during induction therapy (e.g. due to a tumour stenosis) 9. Female patient of childbearing potential (i.e. did not undergo surgical sterilization – hysterectomy, bilateral tubal ligation, or bilateral oophorectomy - and is not post-menopausal for at least 24 consecutive months) with a negative pregnancy test 10. Hematology and biochemistry laboratory results within the limits normally expected for the patient population, defined by the following: • Absolute neutrophil count ≥1500/μl • Platelet count ≥100,000/μl • Leukocyte count >3000/μl • Hemoglobin ≥9 g/dL or 5.59 mmol/l, previous transfusions (>3 days) of erythrocytes are allowed • Total bilirubin ≤1.5 times the upper limit of normal (ULN), in patients with known Meulengracht syndrom ≤3 x ULN • AST ≤ 3xULN in absence of liver metastases, or ≤5xULN in presence of liver metastases • ALT ≤3xULN in absence of liver metastases, or ≤5xULN in presence of liver metastases • Creatinine clearance ≥30 mL/min according to Cockcroft-Gault formula	1. Previous major sugery within the last 28 days before the start of the induction treatment. The implantation of a central venous access (e.g. porth-a cath system) or a diagnostic laparoscopy are allowed. 2. History of other malignant tumours within the last 5 years before start of induction treatment, except basal cell carcinoma or curatively excised cervical carcinoma in situ 3. Known brain metastases 4. Concurrent radiotherapy involving target lesions used for this study. Concurrent palliative radiation for non-target lesions is allowed if other target lesions are available outside the involved field; previous radiotherapy including target lesions must have been finished at least 28 days before start of induction treatment. 5. For patients enrolled before the induction therapy: Previous systemic treatment (i.e. chemotherapy) for metastatic disease 6. Known active HBV, HCV infection or documented HIV infection 7. Serious concomitant disease or medical condition that by judgment of the Investigator renders the patient at high risk of treatment complications 8. Clinically relevant coronary artery disease (NYHA functional angina classification III/IV), congestive heart failure (NYHA III/IV), clinically relevant cardiomyopathy, history of myocardial infarction in the last 3 months or high risk of uncontrolled arrhythmia 9. Female patient pregnant or breast feeding 10. Female patient of childbearing potential (i.e. did not undergo surgical sterilization – hysterectomy, bilateral tubal ligation, or bilateral oophorectomy - and is not post-menopausal for at least 24 consecutive months) not willing to use an adequate method of contraception to avoid pregnancy throughout the study and for up to 26 weeks after the end of treatment. Male patient not willing to use an adequate method of contraception to avoid conception throughout the study and for up to 26 weeks after the end of treatment in such a manner that the risk of pregnancy is minimized. 11. Concurrent treatment with other experimental drugs or participation in another clinical trial with any investigational drug within 60 days prior to start of induction (e.g. one of the allowed standard chemotherapies (see above) with or without additional placebo within a clinical trial is allowed) 12. Chronic diarrhea or short bowel syndrome 13. Known hypersensitivity to S-1, other fluoropyrimidines or platinum compounds. Contraindication to receive S-1 or the polychemotherapy (induction & arm B) chosen for this patient as per current Summary of Product Characteristics. Known DPD deficiency 14. For patients enrolled before the induction therapy: Grade ≥ 2 peripheral neuropathy 15. Known drug abuse/alcohol abuse

### Intervention

**Fig. 1:**
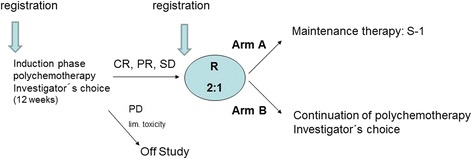
Study design

All patients start with a firstline treatment period of 12 weeks during which a polychemotherapy regimen can be chosen by the local investigator, the following regimens being eligible: cisplatin/capecitabine, cisplatin/5-FU, cisplatin/S-1, FLO/modified Folfox-6, EOX/EOF or FLOT (Table 2). In week 12 a radiological assessment of response will be performed. Patients without tumour progression, with an ECOG-Score of 0 or 1 and without limiting toxicity are randomized in a 2:1 allocation to arm A or B (Fig. 1). Randomization will be stratified by response to primary therapy at time of randomization (CR or PR vs. SD or non-CR/non-PD (in case of non-measurable lesions only)), by the applied polychemotherapy (two-drug vs. three-drug combinations) and by enrollment before vs. after the primary chemotherapy.

**Table 2:** Chemotherapeutical regimens for use during the primary period

Two drug combinations:	Three drug combinations:
Cisplatin/Capecitabine	EOX/EOF
• Cisplatin 80 mg/m^2^, day 1 • Capecitabine 1000 mg/m^2^ twice daily on day 1–14 Each cycle is repeated on day 22.	• Epirubicin 50 mg/m^2^, day 1 • Oxaliplatin 130 mg/m^2^, day 1 • Capecitabine 625 mg/m^2^ twice daily day 1–21 or 5-FU 200 mg/m^2^/day, continuously day 1–21 Each cycle is repeated on day 22
Cisplatin/S-1	FLOT
• Cisplatin 75 mg/m^2^, day 1 • S-1 25 mg/m^2^, twice daily day 1–21 Each cycle is repeated on day 29.	• Docetaxel 50 mg/m^2^, day 1 • Oxaliplatin 85 mg/m^2^, day 1 • Leucovorin 200 mg/mg^2^, day 1 • 5-FU 2600 mg/m^2^ (24-h infusion), day 1 Each cycle is repeated on day 15
Cisplatin/5-FU	
• Cisplatin 75 (−100) mg/m^2^, day 1 • 5-FU 800 (−1000) mg/m^2^/day continuously day 1 to 5 or 1000 mg/m^2^/day cont. day 1–4 Each cycle is repeated on day 22	
FLO	
• Oxaliplatin 85 mg/m^2^, day 1 • Leucovorin 200 mg/m^2^, day 1 • 5-FU 2600 mg/m^2^ (24-h infusion), day 1 Each cycle is repeated on day 15	
Mod. Folfox-6	
• Oxaliplatin 85 mg/m^2^, day 1 • Leucovorin 400 mg/m^2^, day 1 • 5-FU 400 mg/m^2^ (bolus), day 1 • 5-FU 2400 mg/m^2^ (46-h infusion), day 1 Each cycle is repeated on day 15

Patients in arm A receive S-1 monotherapy with a dosage of 30 mg/m^2^ given twice daily per os for 14 days followed by a seven-day rest.

Patients in arm B will continue polychemotherapy with the same regimen as used during the primary phase.

In case of toxicity, chemotherapy doses are reduced according to protocol specifications. Furthermore, in Arm B single agents of the combination regimens can be stopped (e.g. stop of platinum- or taxane-compounds in case of polyneuropathy).

Treatment in the maintenance period will be continued until tumour progression or limiting toxicity.

### Quality of life

Quality of life (QoL) is assessed using the EORTC QLQ-C30 and STO22 questionnaires [22–24]. Baseline QoL will be measured within 14 days before starting the first cycle of firstline treatment. The evaluation will be repeated at the end of primary therapy. During the maintenance therapy and follow-up QoL assessment will be performed at week 4, 9, 13, 18 and 27 after the end of the primary period (including patients who do not continue with maintenance therapy due to tumour progression or toxicity).

### Nutritional assessment

The nutritional state will be evaluated using the Nutritional Risk Screening (NRS) 2002 [25]. Evaluation will be performed before primary chemotherapy, at the end of the primary period, every 9 weeks during maintenance therapy and at the end-of-treatment (EOT).

### Correlative biomarker analyses

The translational part of this trial aims to identify subgroups of patients with a sustained, beneficial response during S-1 maintenance therapy. Blood samples are taken at different time points during the study, polymorphisms of genes related to the fluoropyrimidine metabolism as well as microRNA profiles and cytokine profiles will be measured to identify predictive markers for S-1 maintenance therapy. Formalin-fixed paraffin-embedded tumour tissue will be used for molecular analyses, to correlate clinical efficacy with recently published molecular defined subtypes.

### Statistical considerations

The statistical goal of this randomized phase II trial is to formally assess whether de-escalation with S-1 is not substantially inferior to continuation of polychemotherapy in terms of overall survival. If S-1 can be demonstrated to be non-inferior based on the pre-specified non-inferiority margin and the selected significance level, de-escalation with S-1 can be considered to be promising for further evaluation and subsequent phase III studies, taking into consideration additional potential benefits like decreased toxicity, cost or ease of application. Statistically, the goal is to test whether the ratio of the hazard of death with S-1 divided by the hazard of death with continued polychemotherapy is less than the pre-specified non-inferiority margin.

Selection of a non-inferiority margin is based upon a combination of clinical judgement and statistical reasoning. Literature data suggests that continuation of polychemotherapy will lead to a median overall survival of roughly 8 months (calculated from the time of randomization in week 12) [12, 13, 18]. With this median survival, discussions with clinical experts revealed, the largest increase of the risk of death with S-1 that one is willing to accept for this phase II trial is 33%, if – in addition – safety seems to be in favor of S-1 leading to a non-inferiority margin on the hazard ratio scale of 1.33. The significance level for testing non-inferiority was set to one-sided α = 10%. These choices of both the non-inferiority margin and the significance level can be considered as an initial gatekeeper reflecting the ultimate goal of a phase II design to allow for unbiased evaluation of treatment differences and to support further decision making within the community and to decide whether a phase III trial is justified.

Furthermore, a power of 80% under the alternative hypothesis that both treatment arms are in fact associated with the same underlying hazard rates, i.e. HR = 1 (translating into median overall survival of 8 months in both treatment arms) was pre-specified.

Based on these assumptions and assuming exponential distribution of survival times, 250 observed deaths are required for confirmatory statistical analysis. Since the power of the study is determined by the number of deaths rather than the number of patients, there are a range of sample sizes that could meet the objectives of this trial. Assuming that 10 patients can be randomized per month, with 297 patients (198 treated with S-1 and 99 treated with continued polychemotherapy), the total study duration (accrual plus follow-up) will be about 39 months.

To account for the fact that merely roughly 75% of patients treated with primary chemotherapy will qualify for randomization including patients who were enrolled in the trial after completion of primary therapy, a total of 400 patients will be expected to be enrolled to the trial.

Overall survival (OS) will be defined as the time length between randomization and the date of death from any cause or the date of last follow-up in case of no documentation of death. Kaplan-Meier methods stratified by treatment arm will be applied for estimating the probability of survival over time. For confirmatory analysis of non-inferiority of S-1 a Cox proportional hazards regression model for overall survival with treatment as the only factor and response to primary therapy and polychemotherapy as stratification variables will be fitted. Based on the resulting adjusted hazard ratio for the treatment effect, significant non-inferiority will be claimed if the upper limit of the associated one-sided 90% confidence limit does not exceed the non-inferiority margin θ_0_.

Progression-free survival (PFS) will be defined as the time length between the date of randomization and the date of first disease progression or death (whichever occurs first). Patients alive with no documented progression will be censored at the last documented visit. For statistical analysis, the distribution of progression-free survival time will be described using Kaplan-Meier methods. To adjust for stratification factors used at time of randomization, Cox proportional hazards models will be applied.

When de-escalation with S-1 can be shown to be associated with non-inferior overall survival, assessment whether de-escalation improves QoL compared to continuation of chemotherapy in maintenance phase therapy is of special interest. Therefore, the following statistical approach –exploratory in nature- is foreseen:

Quality of life will be evaluated using the validated EORTC QLQ-C30 questionnaire and the gastric module STO22.

Cumulative distribution of absolute changes (i.e. the proportion of patients who experience every magnitude of change in Global health status/QoL, functional and symptom scales at a time point of interest compared to baseline) will be presented. Furthermore, average scores taken over the first 9 weeks of maintenance treatment and taken over the whole maintenance phase will be calculated for global health status/QoL and all other functional and symptom scales of QoL-questionnaires.

Non-parametric (exact) Wilcoxon-Mann-Whitney tests will be applied for treatment comparisons. To focus on clinically relevant individual changes in QoL measures only, responder analyses showing the rate of patients experiencing a specific change that is meaningful to patients are also pre-specified in the study protocol. To reduce statistical multiplicity issues, a subset of QoL-parameters of special interest is predefined in the study protocol.

### Good clinical practice

This trial is accomplished in conformity with the principals of the Declaration of Helsinki and the Guidelines for Good Clinical Practice in their current revision. The trial will be carried out in keeping with national and international legal and regulatory requirements.

### Current study status

At the time of submitting this manuscript 104 patients have been initially registered, 56 patients have been randomized.

## Discussion

The optimal duration of systemic polychemotherapy for metastatic esophagogastric cancer is unknown. In many clinical trials chemotherapy was given until tumour progression or limiting toxicity, whereas in some other trials treatment was stopped after a pre-defined period of time.

No formal comparison of these two different strategies has been performed.

Given the increasing toxicity rate with a prolonged application of systemic polychemotherapy, the patients’ quality of life could be negatively affected.

During the past years maintenance and de-escalation strategies have been examined in different tumour types like colorectal or lung cancer. Maintenance strategies led to acceptable therapy-related toxicity on the one hand and prolonged the time-to-tumour-progression and overall survival on the other hand [20, 21]. So far and to the best of our knowledge, no published clinical trial has examined yet the role of maintenance therapy in metastatic esophagogastric cancer.

Our study is designed to show non-inferiority of S-1 maintenance therapy in comparison to continuation of polychemotherapy. This comparison was chosen instead of an observational arm without treatment due to the fact that in clinical routine treatment of metastatic esophagogastric cancer is often performed until tumour progression or until limiting toxicity occurs.

In accordance to other clinical trials exploring a platinum-based firstline therapy in metastatic gastric cancer, patients with previous curative surgery with or without perioperative systemic treatment are eligible, if the primary therapy for metastatic disease starts at least six months after the end of perioperative therapy, whereas the subgroup of patients with an early relapse (<6 months after the end of perioperative systemic treatment) is excluded, anticipating a platinum-refractory disease in these patients.

Analysis of changes in the quality of life will be performed during the primary period and maintenance period to examine changes with the Qol in a de-escalated therapeutic setting.

Translational analyses focus on the identification of a subgroup of patients with a sustained benefit of S-1 based maintenance therapy.

This trial is conducted by the Young Medical Oncologists (YMO) Group in cooperation with the Gastric Cancer Working Group of the Arbeitsgemeinschaft Internistische Onkologie (AIO) within the German Cancer Society (Sponsor: AIO-Studien-gGmbH).

## Abbreviations

5-FU: 5-fluorouracil
AEG: Adenocarcinoma of the esophagogastric junction
CR: Complete remission
ECOG: Eastern Cooperative Oncology Group
EORTC: European Organisation for Research and Treatment of Cancer
EOT: End of treatment
Her-2: Human epidermal growth factor receptor 2
NRS: Nutritional risk screening
OS: Overall survival
PFS: Progression-free survival
PR: Partial remission
QLQ: Quality of life questionnaire.
QoL: Quality of life
SD: Stable disease
TTP: Time-to-tumour-progression
VEGFR2: Vascular endothelial growth factor receptor 2
